# Current practices in MRI screening in early onset scoliosis

**DOI:** 10.1007/s43390-024-01033-4

**Published:** 2025-01-22

**Authors:** Austin W. Li, Alexander Chang, Joshua S. Murphy, Ying Li, Benjamin Roye, Christina K. Hardesty, Michael P. Glotzbecker, The Pediatric Spine Study Group

**Affiliations:** 1 Case Western Reserve University School of Medicine, University Hospitals Cleveland Medical Center, Cleveland, OH USA; 2 Pediatric Orthopedic Surgery, George H Thompson Chair in Pediatric Orthopaedics, Rainbow Babies and Childrens Hospital, 11100 Euclid Ave, Cleveland, OH 44106 USA; 3 Department of Orthopaedic Surgery, Emory University, Atlanta, GA USA; 4 Department of Orthopaedic Surgery, University of Michigan, Ann Arbor, MI USA; 5 Pediatric Orthopedics and Spinal Deformity, Columbia University, New York City, NY USA

**Keywords:** Early onset scoliosis, MRI screening practices, Neural axis abnormalities, Syndromic scoliosis

## Abstract

**Purpose:**

Early onset scoliosis (EOS) has traditionally been an indication for MRI because of its association with neural axis abnormalities (NAAs). Because these abnormalities are often clinically silent and concerns regarding sedation in young children are growing, routine MRI for EOS is debated. This study investigates the current practices of EOS MRI screening among surgeons in the Pediatric Spine Study Group (PSSG).

**Methods:**

A survey assessing EOS MRI practices was distributed to the PSSG. The survey presented scenarios that varied in age, curve size, and diagnosis and asked which scenarios would indicate an MRI. Respondents also ranked age, curve progression, etiology, and need for sedation by level of importance when considering to order MRI.

**Results:**

Age and curve progression were ranked as the most important factors when deciding to order MRI. For all non-congenital scoliosis, increased age and curve size were associated with increased rates of MRI among respondents. For idiopathic EOS, more than 60% of respondents would order MRI for patients with curve magnitudes of 45° regardless of age. All respondents would order MRI for congenital EOS before surgery and for EOS caused by neurofibromatosis. For EOS secondary to cerebral palsy, 61% of respondents would order an MRI, and 34% believe that EOS and Prader–Willi syndrome require MRI.

**Conclusion:**

Our results indicate that the MRI screening practices for EOS vary greatly between physicians, as expected. Future research on the prevalence of NAAs in EOS and the clinical outcomes of routine MRI is needed to inform which MRI practices should be standard.

**Supplementary Information:**

The online version contains supplementary material available at 10.1007/s43390-024-01033-4.

## Introduction

Early onset scoliosis (EOS), defined as scoliosis under the age of 10, has traditionally been considered an indication for magnetic resonance imaging (MRI) because of its well-established association with neural axis abnormalities (NAAs), such as syrinxes, tethered cord syndrome, and Chiari malformations [1–4]. Screening for NAAs via routine MRI in EOS allows for earlier diagnosis and therefore earlier treatment, such as by neurosurgery, if necessary. Routine MRI in EOS patients also reduces the prevalence of undiagnosed NAAs, which is important because surgical intervention to correct spinal deformities in patients with unidentified NAAs can lead to neurological complications [5].

The need for routine MRI in EOS patients is, however, widely debated. Not only do previous studies indicate a low prevalence of NAAs in certain EOS patients, but many NAAs identified in EOS patients also tend to be clinically insignificant and do not require treatment, suggesting that MRI in EOS patients might not be necessary [3, 4, 6]. Furthermore, concerns over sedation and anesthesia in young children may have influenced whether MRI is ordered for asymptomatic EOS patients, since prior retrospective studies have suggested potential long-term neurological outcomes associated with early exposure to general anesthesia [7–9].

The primary aim of this study is to better understand current MRI screening practices in EOS by surveying surgeons in the Pediatric Spine Study Group (PSSG). Understanding the current practices of MRI screening, as well as the incidence and clinical implications of NAAs in EOS as determined by other studies, can shed light on the effectiveness of current MRI practices and how they could be modified to improve outcomes and screening efficiency.

## Methods

A survey with clinical scenarios that gauged the age and curve size thresholds that indicate MRI in EOS was distributed to 179 members of the PSSG (see appendix). The PSSG is an international research group of pediatric spine surgeons that has members at 80 different institutions and 11 countries, including the USA, Egypt, Spain, and Hong Kong, among others. The survey presented scenarios that varied by age, diagnosis, and curve size, and respondents stated whether each of these scenarios would indicate MRI. Another primary outcome of the survey was how respondents ranked the four factors of age, curve magnitude, need for sedation, and etiology by level of importance when deciding whether to order MRI. Specifically, respondents were asked to exclusively assign each factor with a number from 1 (most important) to 4 (least important).

### Age and curve magnitude thresholds

To assess the age and curve magnitude thresholds that indicate MRI, the survey asked in a series of yes-or-no questions whether respondents would order MRI for hypothetical EOS patients based on a combination of factors, including patient age (< 1 year old, between 1 and 6 years old, or > 7 years old), curve magnitude (major curve with Cobb angle of 20° or 45°), and etiology. The etiologies included in the survey were presumed idiopathic EOS without kyphosis, neuromuscular scoliosis (non-ambulatory cerebral palsy), syndromic scoliosis, and congenital scoliosis. Our survey also specifically asked whether respondents believed patients with cerebral palsy (CP) and EOS required MRI, and whether respondents would order MRI for all congenital EOS patients before surgery. Questions regarding congenital scoliosis specified a type of congenital scoliosis (congenital isolated hemivertebrae, multiple congenital vertebral anomalies, or congenital kyphosis). Included in each scenario was the patient’s need for sedation. The frequency of respondents who would order MRI in each of these scenarios was recorded as the primary outcome for this portion of the survey.

### Syndromic diagnosis

To assess which syndromic diagnoses warrant MRI regardless of age, curve pattern, or need for sedation, the survey provided a list of common syndromes associated with EOS and asked respondents to answer accordingly. The syndromes included were Marfan syndrome, neurofibromatosis, Prader–Willi syndrome, osteogenesis imperfecta (OI), skeletal dysplasia, and mucopolysaccharidoses. Respondents were also given an “other” option, in which they could freely enter syndromic diagnoses that they believed indicated MRI.

### Curve size threshold and additional MRI practice assessments

For patients with idiopathic EOS without kyphosis, the respondents were asked to input the Cobb angle at which they would order MRI regardless of age or need for sedation. Lastly, the survey asked discrete questions that assessed general MRI practices. These questions are listed below:

*Do you have a rapid MRI protocol?*

*Do you get an MRI, regardless of age/etiology, if you initiate treatment (brace, cast, or surgical)?*

## Results

The survey received 41 responses (response rate = 23%). Of the 41 respondents, 3 were neurosurgeons and 38 were orthopedic surgeons. The survey yielded responses from the USA (31), Canada (4), UK (2), Hong Kong (2), Finland (1), and New Zealand (1), and the respondents stemmed from a total of 35 different medical institutions.

Among these responses, age and curve magnitude were the two most important factors (mean = 2.2) when deciding whether to order MRI (Table 1). Etiology was the third highest ranked factor (mean = 2.49), and need for sedation had the lowest ranking (mean = 3.12).

**Table 1:** Ranking of influential factors when ordering MRI (lower rank indicates higher importance)

Factor	Mean rank	St. dev
Curve magnitude	2.2	1.03
Age	2.2	1.1
Etiology	2.49	0.95
Need for sedation	3.12	1.17

### Age and curve magnitude thresholds

The survey results for the age and curve magnitude thresholds are presented in Table 2. Respondents generally believed that increased curve magnitude and age indicated MRI. Despite this trend, some differences were noted between diagnoses.

**Table 2:** Physician perspectives on MRI usage depending on age, curve size, and diagnoses

Condition and curve size	< 1 year old	1–6 years old	> 7 years old
Presumed idiopathic early onset scoliosis (without kyphosis)			
20° Curve	*12%*	*22%*	**95%**
45° Curve	**68%**	**88%**	**95%**
Neuromuscular scoliosis (non-ambulatory CP)			
20° Curve	*13%*	*13%*	*17%*
45° Curve	42%	50%	54%
Syndromic scoliosis (Marfan, NF, Prader–Willi, OI, skeletal dysplasia, etc.)			
20° Curve	*14%*	*22%*	*30%*
45° Curve	**62%**	**89%**	**92%**
Congenital scoliosis			
Isolated hemivertebrae (operative)	–	**95%**	**95%**
Isolated hemivertebrae (non-operative)	37%	*14%*	**74%**
Multiple congenital vertebral ± rib anomalies (operative)	–	**100%**	**100%**
Multiple congenital vertebral ± rib anomalies (non-operative)	47%	**63%**	**89%**
Congenital kyphosis (operative)	–	**100%**	**100%**
Congenital kyphosis (non-operative)	47%	58%	**89%**

Percentages greater than 60% are bolded, whereas percents less than 30% are italicized. *CP* cerebral palsy, *OI* osteogenesis imperfecta

61% of respondents agreed that patients with CP do *not* require MRI

For presumed idiopathic EOS without kyphosis and syndromic scoliosis, most respondents would order an MRI regardless of age if the curve size was 45°. More specifically, 68%, 88%, and 95% would order MRI for idiopathic EOS patients < 1 year of age, between 1 and 6 years of age, and > 7 years of age, respectively. However, for curves of 20°, most respondents would not order an MRI for patients with presumed idiopathic EOS unless they were > 7 years of age, for which 95% would order MRI (Table 2).

In contrast, 61% of respondents agreed that non-ambulatory CP patients with EOS do *not* require MRI. Less than 20% of the respondents would order MRI for patients with CP for all age groups if the curve size was 20°. While more respondents would order MRI for patients with CP and curve sizes of 45°, only 54% of total respondents would order MRI even for patients > 7 years of age (Table 2).

For patients with congenital scoliosis and isolated hemivertebrae undergoing operative treatment, 95% of respondents would order an MRI regardless of age. Likewise, 100% of respondents would order an MRI for congenital scoliosis with kyphosis undergoing operative treatment. For patients undergoing non-operative treatment of multiple congenital vertebral abnormalities with or without rib anomalies and congenital kyphosis, increased age was associated with increased rates of ordering an MRI among respondents. For example, 63% of respondents would order MRI for patients with this diagnosis if the patient were between 1 and 6 years of age, whereas 89% would order MRI for patients > 7 years of age. Interestingly, for non-operative isolated hemivertebrae, more respondents would order an MRI for patients < 1 year of age (34%) than for patients between 1 and 6 years of age (14%). Congenital scoliosis with non-operative isolated hemivertebrae was the only diagnosis in this study that yielded higher MRI rates for patients < 1 year of age than for patients between 1 and 6 years of age.

### Syndromic diagnosis

The results for MRI frequency among respondents for specific syndromes are shown in Table 3. All respondents agreed that EOS with neurofibromatosis is an indication for MRI, and 80% of respondents agreed that EOS with Marfan syndrome also requires MRI. Only 34% of respondents believed that patients with EOS and Prader–Willi syndrome should routinely undergo MRI. One respondent indicated through the “other” option that some unlisted genetic disorders associated with spinal deformity would warrant MRI. This respondent did not name any specific genetic disorders in their answer.

**Table 3:** Frequencies of YES to MRI based on syndromic diagnoses

Syndromic diagnosis	Frequency of YES for MRI	Percentage
Neurofibromatosis	41	100%
Marfan	33	80%
Skeletal dysplasia	31	76%
Mucopolysaccharidoses	31	76%
Osteogenesis imperfecta	25	61%
Prader–Willi	14	34%
Other	1	2%

### Curve size threshold and additional MRI practice assessments

The mean curve magnitude that warrants MRI in presumed idiopathic EOS without kyphosis was 32° (± 11°), regardless of age or need for sedation. Among the 41 respondents, 56% have a rapid MRI protocol, and 46% get an MRI regardless of age/etiology if treatment were initiated.

## Discussion

The routine use of MRI in EOS has become a widely debated topic among pediatric spine surgeons. EOS has traditionally been considered an indication for MRI because of its well-established association with NAAs [1–4, 10]. A previous international cohort study by Williams et al. investigated EOS patients who underwent pretreatment MRI and found that roughly 24% of EOS patients had abnormal MRI findings [4]. Previous studies have argued that this increased prevalence of NAAs necessitates routine MRI in EOS, especially since some NAAs require neurosurgical intervention [10]. Furthermore, some argue that early identification of NAAs with routine MRI is important as they may indicate an increased risk for surgical complications if undiagnosed [4].

More recent studies suggest that most NAAs identified with routine MRI in EOS have few or no clinical implications and often do not require specific intervention, making MRI unnecessary [6]. Concerns over the need for sedation also challenge whether routine MRI should be performed. Some studies have suggested an association between early exposure to general anesthesia and long-term neurocognitive deficits in adulthood, such as learning, motor function, memory, and behavior [11, 12]. These associations have only been demonstrated in non-human primates and rodents, and it remains unclear whether they exist in humans as well.

The present study investigated current MRI practices in EOS among pediatric orthopedic surgeons and neurosurgeons in the PSSG. Our results found that age and curve size were the most important factors for PSSG members when deciding whether to order an MRI. Despite this, the previously mentioned international cohort study by Williams et al. found little correlation between age or curve magnitude with abnormal MRI findings [4]. Therefore, future research should continue to investigate the prevalence of NAAs in EOS for different age groups and curve sizes to determine how MRI screening practices should be affected by these factors.

Our study shows that PSSG members believe that the need for sedation is a relatively unimportant factor in determining whether to order an MRI. Current data on the long-term implications of sedation in young children support this ranking, as several studies have shown little to no evidence of increased risk of neurocognitive disabilities after early exposure to general anesthesia [8, 13]. Furthermore, recent development of rapid MRI protocols may swing the pendulum toward more MRIs in younger patients. Only 56% of respondents in the current study currently use such a protocol. As more centers adopt these protocols, we may expect an increased use of MRI in certain populations.

One of the primary motivations for performing routine MRI in EOS is to prevent undiagnosed NAAs, which can lead to surgical complications in patients requiring operative treatment. The results of this study indicate that 95–100% of respondents would order an MRI for all congenital EOS patients requiring operative treatment before surgery. Similarly, most respondents felt an MRI was warranted in larger curve sizes associated with other diagnoses besides CP, suggesting that an MRI would be available for curves that require surgical intervention. This might support the perspective that not all EOS patients require MRI and that need for surgical correction should be a major indication for MRI.

The responses to our survey also illustrate that EOS etiology largely influences MRI practices. Our study found that 100% of respondents would order a MRI for all patients with neurofibromatosis regardless of age or curve magnitude, and that most respondents would do the same for patients with Marfan syndrome, OI, skeletal dysplasia, and mucopolysaccharidosis. However, the prevalence of NAAs in patients with Marfan syndrome, OI, skeletal dysplasia, and mucopolysaccharidosis has yet to be determined, and thus it is unclear whether routine MRI to identify NAAs is truly needed for these diagnoses. It is also important to note that for Marfan syndrome and neurofibromatosis, MRI is often performed to identify other abnormalities, such as dural ectasia, and not NAAs. This practice may explain why most of our respondents would order MRI for Marfan syndrome and neurofibromatosis despite the unclear association between these diagnoses and NAAs.

The study by Williams et al. also found that patients with neuromuscular EOS had the highest incidence of NAAs compared to EOS patients with other etiologies [4]. Despite this finding, 61% of the respondents to our survey agreed that non-ambulatory CP patients with EOS do *not* require MRI. While the study by Williams et al. did not specify between CP and other types of neuromuscular EOS, their findings perhaps argue for more frequent MRI screening in neuromuscular EOS. Future research should continue to investigate the prevalence of NAAs in patients with EOS and CP to determine whether the current practices of MRI screening for EOS and CP patients should be modified.

This study has several limitations. First, the survey had a low response rate, which means the results may not accurately represent widespread MRI practices. However, the geographic diversity of the respondents may help combat this potential sampling bias, as our respondents stemmed from over 30 different institutions in 6 different countries. Another limitation of this study was the limited assessment of curve magnitude. The survey only distinguished between two curve sizes (20° and 45°). To better determine how curve magnitude affects MRI practices in EOS, future surveys should present scenarios that have a wider range of curve magnitudes or ask respondents to state which curve magnitudes would warrant MRI for specific clinical scenarios. Similarly, our survey did not evaluate MRI practices for specific syndromes, such as Marfan or Prader–Willi, and instead grouped syndromic scoliosis into one category. Therefore, it is unclear whether the practices determined for “syndromic scoliosis” in our survey are applicable to all syndromes or represent the practices of a select few. Therefore, future research should continue to investigate the MRI practices for specific syndromes associated with EOS.

## Conclusion

This study aimed to identify the current MRI screening practices in EOS among an international group of pediatric spine surgeons. The results showed that age, diagnosis, and curve magnitude all influence whether MRI is ordered. Despite these trends, the responses were varied, revealing largely unstandardized MRI screening practices. Future research should continue to investigate the prevalence and clinical implications of NAAs in patients with EOS, as well as the relative risk of rapid MRI protocols, so that future MRI practices can be streamlined to improve clinical outcomes.The authors confirm that the data supporting the findings of this study are available within the article and its supplementary materials.

## Supplementary Information

Below is the link to the electronic supplementary material.

Supplementary file1 (DOCX 22 kb)
