# Supplementary material for: Current practices in MRI screening in early onset scoliosis
Source: Spine Deform. 2025 Jan 22;13(3):961–6. doi: 10.1007/s43390-024-01033-4 (PMC12021731; doi:10.1007/s43390-024-01033-4)
Supplement: Supplementary file 1 — Supplementary file1 (DOCX 22 kb) [file 43390_2024_1033_MOESM1_ESM.docx]

**Appendix:**

**Survey: Best Practices in MRI screening in children with Early Onset Scoliosis**

Early Onset Scoliosis (age < 10) has traditionally been considered an indication for MRI. However, concerns over sedation/anesthesia in young children may have influenced practice in patients that do not exhibit any concerning signs or symptoms. Others may feel that some diagnoses that are known to be commonly associated with EOS do not warrant an MRI. Therefore, the following survey aims to identify age and curve size/pattern thresholds of members of the PSSG.

Do you have a rapid screening protocol? (Y/N)

In child with normal neurologic exam that you are treating nonoperatively rate the following factors in level of importance:

Age

Curve progression

Etiology

Need for sedation

Do you get an MRI regardless of age/etiology if you initiate treatment? (Y/N)

In a patient with Early Onset Scoliosis with a normal neurologic exam, without unexpected rapid progression, and no skin/exam findings concerning for spinal dysraphism (assuming these would be indications for MRI), would you order an MRI for the following patients? (Answer all Y or N)

Presumed Idiopathic Early Onset Scoliosis (without kyphosis):

<1 year old with 20 degree curve

<1 year old with 45 degree curve

2 year old with 20 degree curve

2 year old with 45 degree curve

4 year old with 20 degree curve

4 year old with 45 degree curve

>7 year old with 20 degree curve (doesn’t require sedation)

>7 year old with 45 degree curve (doesn’t require sedation)

What threshold curve size would warrant an MRI regardless age or need for sedation? Cobb Angle >____

Neuromuscular scoliosis (nonambulatory CP)

<1 year old with 20 degree curve

<1 year old with 45 degree curve

2 year old with 20 degree curve

2 year old with 45 degree curve

4 year old with 20 degree curve

4 year old with 45 degree curve

>7 year old with 20 degree curve

>7 year old with 45 degree curve

I don’t think patients with CP and EOS need an MRI (Y/N)

Does kyphosis influence your likelihood of getting MRI at smaller curve or younger age? (Y/N)

Congenital scoliosis:

<1 year old with isolated hemivertebrae

<1 year old with multiple congenital vertebral +/- rib anomalies

<1 year old with congenital kyphosis

2 year old with Isolated hemivertebra

2 year old with multiple congenital vertebral +/- rib anomalies

2 year old with congenital kyphosis

4 year old with isolated hemivertebrae (operative)

4 year old with isolated hemivertebrae (non-operative)

4 year old with multiple congenital vertebral +/- rib anomalies (operative)

4 year old with multiple congenital vertebral +/- rib anomalies (non-operative)

4 year old with congenital kyphosis (operative)

4 year old with congenital kyphosis (non-operative)

>7 year old with isolated hemivertebrae (no sedation needed) (operative)

>7 year old with isolated hemivertebrae (no sedation needed) (non-operative)

>7 year old with multiple congenital vertebral +/- rib anomalies (no sedation needed) (operative)

>7 year old with multiple congenital vertebral +/- rib anomalies (no sedation needed) (non-operative)

>7 year old with congenital kyphosis (no sedation needed) (operative)

>7 year old with congenital kyphosis (no sedation needed) (nonoperative)

Syndromic scoliosis (Marfan, NF, Prader-Willi, OI, skeletal dysplasias, etc.)

<1 year old with 20 degree curve

<1 year old with 45 degree curve

2 year old with 20 degree curve

2 year old with 45 degree curve

4 year old with 20 degree curve

4 year old with 45 degree curve

>7 year old with 20 degree curve

>7 year old with 45 degree curve

I don’t think patients with a syndromic diagnosis and EOS need an MRI (Y/N)

Does kyphosis influence your likelihood of getting MRI at smaller curve or younger age? (Y/N)

What syndromic diagnoses do you feel need an MRI in EOS? (check all that apply)

Marfan

NF

Prader-Willi

OI

Skeletal dysplasia

Mucopolysaccharidoses

Other (free text):
